# Parkinson’s Disease, SARS-CoV-2, and Frailty: Is There a Vicious Cycle Related to Hypovitaminosis D?

**DOI:** 10.3390/brainsci13040528

**Published:** 2023-03-23

**Authors:** Sara Palermo, Mario Stanziano, Anna Nigri, Cristina Civilotti, Alessia Celeghin

**Affiliations:** 1 Department of Psychology, University of Turin, 10124 Turin, Italy; 2 Neuroradiology Unit, Diagnostic and Technology Department, Fondazione Istituto di Ricovero e Cura a Carattere Scientifico (IRCCS) Istituto Neurologico Carlo Besta, 20133 Milan, Italy; 3 Neurosciences Department “Rita Levi Montalcini”, University of Turin, 10126 Turin, Italy

**Keywords:** Parkinson’s disease, COVID-19, frailty, hypovitaminosis D, α-synuclein origin site and connectome (SOC) model, neuroimaging

## Abstract

The literature has long established the association between aging and frailty, with emerging evidence pointing to a relationship between frailty and SARS-CoV-2 contagion. The possible neurological consequences of SARS-CoV-2 infection, associated with physical and cognitive frailty, could lead to a worsening of Parkinson’s disease (PD) in infected patients or—more rarely—to an increase in the Parkinsonian symptomatology. A possible link between those clinical pictures could be identified in vitamin D deficiency, while the whole process would appear to be associated with alterations in the microbiota–intestine–brain axis that fall within the α-Synuclein Origin site and Connectome (SOC) model, and allow for the identification of a body-first PD and a brain-first PD. The model of care for this condition must consider intrinsic and extrinsic variables so that care by a multidisciplinary team can be successfully predicted. A multidimensional screening protocol specifically designed to identify people at risk or in the early stages of the disease should begin with the investigation of indices of frailty and microbiota–intestine–brain axis alterations, with a new focus on cases of hypovitaminosis D.

## 1. Introduction

Over the past years, attention to the possible effects of SARS-CoV-2 on the brain has greatly increased. The neurologic and neuropsychologic manifestations of SARS-CoV-2 infection have been described in single case reports, retrospective series of sparse cases, prospective studies and reviews. Evidence has demonstrated that COVID-19 is neuroinvasive, neurotropic, and neurovirulent [1].

Due to the loss of the sense of smell, the olfactory nerve is suspected to be the primary route of neuroinvasion via hematogenous diffusion. Additional neuropsychological symptoms, such as headaches, seizures, confusion, memory problems, delirium and psychosis, were reported in the description of the phenomenon [1]. Referring to neurological disorders, caused by an immune reaction against COVID-19, acute disseminated ataxia, pyramidal signs, impaired consciousness, encephalitis, encephalomyelitis, acute hemorrhagic myoclonus, cytokine release and mononeuritis, myositis, cerebral vasculitis, transverse myelitis, and Guillain–Barre syndrome were observed [1]. Many of these neurological diseases have immunological pathogenesis, accelerated by immunosenescence and inflammaging [2] and possibly exacerbated or triggered by SARS-CoV-2.

In line with this, Parkinson’s disease (PD) or Parkinsonism has been described after infections with viruses, such as the Epstein–Barr virus, hepatitis C virus, HIV, influenza A virus, Japanese encephalitis virus, varicella zoster virus, or West Nile virus [3,4]. Therefore, the hypothesis that SARS-CoV-2 may have even longer-term effects on the brain and lead to an increase in cases of Parkinson’s disease, as occurred in the years following the Spanish flu [3,5,6], has been put forward. It may be possible that the incomplete understanding of the phenomenon is caused by the absence of other potentially significant predictors or mediational factors in the prodromal evaluation of clinical data. Among all, one element associated with possible effects on neurodegenerative processes that has attracted attention over the years is frailty [7,8,9]. 

Frailty identifies a condition of risk and vulnerability characterized by an unstable balance in the face of negative events. Stressful conditions can negatively impact the physical and mental well-being of the elderly, due to reasons related to aging and intercurrent diseases [2]. That condition is associated with a worsening health and an increased risk of hospitalization, institutionalization, falls and death [2]. The presence of a frail condition, therefore, exposes one to the risk of developing a disabling condition in the short term.

In this specific case, two possible scenarios can be considered. As a result of SARS-CoV-2 infection, structural alterations in the basal ganglia may result in a variety of symptoms, including Parkinsonism [10,11]. Second, infection may reveal asymptomatic PD or exacerbate disease progression. Considering this second eventuality, it might be interesting to investigate what cofactors might make these individuals more prone to PD when infected with COVID-19. 

A cog element in the triadic interaction (SARS-CoV-2, frailty and PD) may lie in vitamin D levels. Hypovitaminosis D appears to be associated with the risk of SARS-CoV-2 infection, the incidence and severity of COVID-19 and mortality [12]. Hypovitaminosis D is also common in several age-related chronic diseases such as osteoporosis, sarcopenia and cognitive impairment, and the incidence of hypovitaminosis D increases with increasing age [13]. Importantly, vitamin D deficiency has also been associated with the risk of PD and motor severity [14]. The aim of this contribution is to provide some suggestions on all these aspects.

## 2. COVID-19 and Parkinson’s Disease: “Is There an Unexpected Relationship?”

There is evidence that the COVID-19 virus can cause damage to the brain cells, potentially triggering a neurodegenerative process [3,5]. On average, the loss or reduction of the sense of smell has been reported in three out of four people infected with SARS-CoV-2 [5]. Although the loss of smell may not merit particular concern, it actually tells us a lot about what is going on inside the body: there is acute inflammation in the olfactory system [5,15]. Angiotensin-converting enzyme 2 (ACE2) dysregulation is implicated in the entry of SARS-CoV-2 into human host cells. Olfactory neurons are supported by ACE2 receptor-positive sustentacular cells. Salt ions in the mucus are delicately balanced by these cells, and if this balance is upset, neuronal signals to the brain may cease, which can lead to olfaction perturbation. These cells also provide metabolic and physical support to the finger-like cilia that line the olfactory neurons responsible for odors detection [16]. 

There is no doubt that an ACE2 imbalance contributes to the core pathologies of PD and COVID-19, including aberrant inflammatory responses, oxidative stress, mitochondrial dysfunction and dysregulation of the immune system. Moreover, alpha-synuclein-induced dopaminergic degeneration, gut–brain axis dysregulation, blood–brain axis disruption, autonomic dysfunction, anxiety, depression and hyposmia are all associated with ACE2 dysregulation in PD [17]. It is precisely the loss of smell that could be a new way of detecting the risk of developing Parkinson’s disease at an early stage, as it occurs in 90% of people who contract the disease and are still in the preclinical/early phase, which is about a decade before the appearance of motor symptoms [18]. Considering that the opportunity to adopt neuroprotective therapies that achieve the desired effect is lost by waiting until the onset of the motor phase of Parkinson’s disease, this event could open up the adoption of integrated treatments earlier than the standard period.

Research investigating possible relationships between COVID-19 infection and PD has yielded mixed results [6]. What is known is that Parkinson’s disease patients do not have a higher risk of becoming infected [19] but, once infected, an increased risk of more serious infection is possible [6]. More recently, it has been shown that the aggregate prevalence of COVID-19 infection in PD cases is 5%, in addition to hospitalization and mortality rates of 49% and 12% [20]. Older and more chronic PD patients appear to be particularly susceptible to infection, with a significantly higher mortality rate (40%) and with a particular risk of death for those undergoing advanced therapies, such as deep brain stimulation or levodopa infusion therapy [21]. Individuals with PD-MCI were more likely to experience PD-specific rather than cognitive symptoms after quarantine [22], and in PD COVID-19-confined patients, the cognitive and behavioral functions deteriorated more rapidly than in subjects without motor impairment [22]. Moreover, the infection induced significant worsening of motor performance, motor disability, and daily living experiences, including increased fatigue [22,23]. Although many PD patients present with typical COVID-19 symptoms, some atypical PD patients present with an isolated worsening of the Parkinsonian symptoms, requiring increased dopamine replacement therapy and presenting worse outcomes [6]. The worsening of the PD symptomatology, including gait dysfunction and risk of falls, may be attributed to the reduced mobility, stress, anxiety and isolation during the pandemic, which could have negative consequences; however, SARS-CoV-2 infection has also been linked to secondary neurodegeneration or may have an effect on dopaminergic neurotransmission [24]. 

The mechanism that might compromise the nigrostriatal dopaminergic nervous systems during COVID-19 is unclear [25,26]. A *susceptible genetic make-up* might make patients vulnerable to immunologically mediated mitochondrial damage and neuronal oxidative stress [25,26]. Another hypothesis could be that the virus causes *inflammation through microglial activation*, contributing to protein aggregation and neurodegeneration [27]. Indeed, the exosomal cargo of the SARS-CoV-2 virus may be capable of promoting neurodegenerative and neuroinflammatory cascades in the brain as it travels from the periphery to the brain. This cascade may lead to the development of Parkinsonism and Parkinson’s disease [28]. Finally, the *multiple stroke hypothesis* favors the combination of toxic stress and inhibition of neuroprotective responses leading to neuronal death [29]. Immune activation in the olfactory system could therefore lead to α-synuclein misfolding and the development of Parkinson’s disease [30,31]. This mechanism is supported by post-mortem studies, which show increased levels of pro-inflammatory cytokines (such as tumor necrosis factor [32] and interleukins IL1 and IL6 [33]). In addition, patients with Parkinson’s disease have an elevated CSF antibody response to seasonal coronaviruses, compared to healthy controls of the same age [34]. 

### Neuroimaging Evidence

The brain inflammation and damage to neuronal circuits seen in patients who died from COVID-19 are similar to those seen in Alzheimer’s and Parkinson’s patients [35]. Extensive cellular perturbations have been observed, predicting how choroid plexus barrier cells perceive and transmit peripheral inflammation in the brain and showing how peripheral T-cells infiltrate the parenchyma [35]. In addition, genes related to cognition, schizophrenia and depression would be more frequently activated in the brains of COVID-19 patients [35].

Reports of neuroimaging data of patients who developed Parkinsonism have followed one another over time. Mendez-Guerrero and colleagues [36] reported a case in which DaT-SPECT confirmed a bilateral decrease in presynaptic dopamine uptake that asymmetrically involved both putamen regions. Cohen and collaborators [37] confirmed this type of alteration in another patient. PET scanning showed a decrease in 18F-FDOPA uptake in both putamen regions, more evident on the left side [37]. This was also found in a non-postencephalitic case [38], for which a decreased dopamine transporter density on the left putamen (even if more evident in the mid-putamen) was clearly traceable.

Morassi and colleagues [39] found that the FDG-PET/CT results resembled those observed in post-encephalitic Parkinsonism, with cortical hypo-metabolism associated with hyper-metabolism in the brain stem, mesial temporal lobes and basal ganglia. Moreover, hypermetabolic brain areas correlated with brain regions showing increased cortical thickness, suggesting their involvement during the inflammatory process [39]. 

## 3. Physiological Aging: From Frailty to Susceptibility to Neurodegenerative Diseases and Viral Infection

Older adults are more prone to frailty and disease onset due to an impaired crosstalk between the innate and the adaptive arms of the immune system and to inflammaging [2,40,41]. 

In addition to immunosenescence and inflammaging, a number of mechanisms contribute to COVID-19 infection risk and severity, such as a cytokine storm and a reduced gut microbiota diversity, which causes frail elderly to have a weak immune system [42]. Importantly, a change in the gut microbiota is better indicated by frailty than chronological age [43]. 

The increase in inflammatory cytokines is related with sarcopenia and muscle weakness, possibly due to prolonged immobility during hospitalization or confinement during lockdown. In addition, a common feature of infectious diseases is a decrease in calorie and nutritional intake, which can, again, negatively impact the health of muscles, bones and joints [44,45]. Eventually, this clinical picture can lead to a decline in multiple systems’ function [46], with a disrupted homeostasis, accelerated aging and age-related diseases [47]. 

The neurotrophic properties of the virus and the infection-induced sustained pro-inflammatory state facilitate neurodegenerative processes through increased beta-amyloid deposition and microglia activation [48,49,50,51], leading to Alzheimer’s and Parkinson’s disease, which are the two most common pathological manifestations. Indeed, a systemic inflammatory status and increased oxidative processes could explain the impact of pneumonia on cognition. In addition, pneumonia-related hypoxia is also linked to neurodegeneration and cerebrovascular injury, which predispose individuals to cognitive impairment and major neurocognitive disorders [52]. As a result, frail older adults are more likely to suffer adverse outcomes and death from SARS-CoV-2 infection [42] and experience a less effective COVID-19 vaccination constellated with more side effects [2,42]. 

The incidence rates of physical-cognitive decline and frailty increase with the aging population and with the presence of neurodegenerative diseases. Affecting more than 1% of the population over the age of 60 and 5% of people over the age of 85, PD is closely linked to both aging and frailty [53]. Precisely, the association between weight loss (one of the clinical biomarkers of frailty according to Fried’s biomedical model), poor nutritional status, motor complications and PD progression is a frequent yet under-recognized complication in Parkinson’s disease [54,55]. Ahmed and coauthors [56] found that one-third of patients with optimally treated PD met the criteria for frailty, a prevalence five-fold higher than expected in a comparable elderly population; moreover, the number of diagnostic components positive for frailty increased with the severity of PD. Indeed, an association between frailty and PD disease duration, motor impairment, postural instability/gait difficulty and total daily levodopa dose was observed [57]. Moreover, frailty appears to be associated with a mild behavioral impairment, with potential effects on impulse dyscontrol [58], which has previously been associated with executive-metacognitive dysfunction in Parkinson’s disease [59,60]

PD patients can present a progressive decline in independence and quality of life, which is similar to that observed in frail older adults; however, frailty and PD are clinically distinct entities with a differing underlying pathophysiology [56,57]. The pandemic aggression has precipitated both the incidence of Parkinsonian symptoms and the progression to PD and frailty [61,62]. This allows us to hypothesize an interacting triad toward vulnerability, disability and chronicity of disease. Looking for studies exploring this existing relationship (PubMed searched until December 2022), only six articles were found to be indexed in PubMed [63,64,65,66,67,68]. Collectively, those studies conclude that the presence of previous mental or physical frailty caused by PD generated a worse prognosis, with an inauspicious outcome due to SARS-CoV-2 infection in a high number of cases [64,67,68]. Moreover, the COVID-19 pandemic has exacerbated behavioral and mood changes associated with PD-related frailty [63,65,68]. 

## 4. A Further Hint: Vitamin D

Vitamin D is a key regulator of development and maturation of all immune lineages. It also contributes to innate immunity through its antimicrobial action, modulation of adaptive response and induction of tolerance [12]. More specifically, 25-OH Vitamin D is able to induce the expression of cathelicidin and β-defensin 2, proteins with (*in*)direct antimicrobial efficacy through the stimulation of chemotaxis in immune system cells, inducing the expression of pro-inflammatory cytokines and resulting in the removal of infected cells in the respiratory tract [69]. 

### 4.1. Vitamin D, SARS-CoV-2 Infection Risk and Severity 

The hypothesis of the role of vitamin D in the susceptibility to SARS-CoV-2 infection stems from the observation of the high prevalence of hypocalcemia (50%) among hospitalized patients during the Ebola (2016) and SARS (2003) epidemics [70]. Up to 80% of COVID-19 patients hospitalized in Italy during the first wave of the pandemic reported a reduction of the quantity of calcium in laboratory exams ([Ca^2+^] < 1.18 mmol/L). Free calcium is required for virus–cell interactions (via the spike protein and ACE2), viral replication and the inflammatory response to the infection. The association between vitamin D status and infection risk could therefore be, at least in part, consequent to the deregulation of (phospho)calcium homeostasis [71]. The fact that calcium is critical in the infection process is also demonstrated by the fact that the pharmacological blockade of L-type calcium channels slows the rate of replication of porcine deltacoronavirus [72]. Moreover, an increased risk of SARS-CoV-2 infection, with an OR of 1.43 (95% CI of 1.00–2.05), was found in association with deficient values of vitamin D (25(OH)D) (vitamin D deficiency is defined as less than 20 ng/mL (50 nmol/L), while insufficiency corresponds to 21–29 ng/mL (52.2–72.5 nmol/L)) [73].

Furthermore, comorbidities and aging are involved. Therefore, it is impossible to determine whether micronutrient deficiency contributes to the SARS-CoV-2 infection risk or, rather, reflects (or is consequent to) a pathophysiological condition that itself increases the infection risk. Chronic hypovitaminosis D may, however, predispose to comorbidities and thus indirectly influence disease severity: for example, advanced age and obesity are associated with vitamin D deficiency and a more severe COVID-19 course [74]. Despite the fact that lower levels of vitamin D appear to be associated with more severe COVID-19 symptoms and an increased risk of hospitalization, the association with other clinical outcomes—such as mechanical ventilation, intensive care unit admissions and mortality—is unclear [75]. 

### 4.2. Vitamin D: Common Element in the Continuun between Aging, Frailty and Parkinson’s Disease

In the human body, vitamin D is produced endogenously when the skin is exposed to sunlight, whereas exogenous sources include certain natural foods and supplements. The vitamin D intake in Europe is generally low, between 2 and 3 g per day [76]. It has been found that there is a north–south gradient in the 25(OH)D status, with more-northern countries (Sweden and Finland) having higher percentage values than southern countries (Spain and Italy) [77]. Hypovitaminosis D is particularly prevalent in the elderly population, particularly among institutionalized and community-dwelling elderly [21,78], as well as among those over 80 [79]. Lower levels of vitamin D are associated with reduced sun exposure and reduced skin synthesis capacity, leading to lower levels of cholecalciferol [80]. In addition, renal function efficiency decreases with aging, resulting in a reduction in the activity of the 1-hydroxylase enzyme that converts 25(OH)D into calcitriol, whose levels in the elderly are inversely related to serum creatinine levels and glomerular filtration rates [81]. A reduction in the level of the VDR receptor (specific for 25(OH)D) within the skeletal muscle system of the elderly and the progressive decline of intestinal mucosal sensitivity to calcitriol may also contribute to vitamin D deficiency [80].

Many geriatric pathological conditions, including bone fragility and fractures, sarcopenia, neoplasms, cardiovascular disease and depression, are related to hypovitaminosis D [80]. The picture described is consistent with the frailty syndrome. There is no doubt that vitamin D has a role to play in frailty, as both animal models and large population-based studies have shown that low serum vitamin D levels are associated with a higher risk of frailty in humans [82]. Based on a meta-analysis [83], the OR of frailty for the lowest versus the highest level of vitamin D can reach 1.27 (95% CI = 1.17–1.38, I2 = 59%), with a low vitamin D level significantly associated with the frailty risk in women. Indeed, postmenopausal women are prone to sarcopenia, which could result in functional impairment, disability, and fractures [84]. Subsequent quantitative analyses confirmed significant differences when comparing frail and pre-frail subjects [85]. Additionally, 25(OH)D concentrations were significantly associated with lower gait speed and impairment in the Timed Up and Go test, two measures of physical performance associated with frailty in the elderly [86]. Finally, a significant relationship was observed between physical frailty and serum vitamin D concentration in predicting cognitive frailty [87].

The role played by hypovitaminosis D in PD patients compared to a control group was ascertained over two decades ago [88]. Studies found a high prevalence of hypovitaminosis D in Parkinson’s disease individuals [89,90], elucidating the inverse association between blood 25-hydroxyvitamin D concentrations and the incidence of PD [91]. This is due to a deterioration in the gastrointestinal function, which is significantly more prevalent in people with “early PD” [89,92]. Hypovitaminosis D appears to be associated with disease severity and progression, but not with PD onset age or illness duration. Furthermore, reduced vitamin D levels in PD patients have been linked to a higher risk of falling. Less is known regarding vitamin D impact on the cognitive function and other nonmotor symptoms [14,88].

Importantly, vitamin D3 supplementation has been found to improve both motor and non-motor PD symptoms, as well as the overall quality of life of PD patients [93]. Boosting vitamin D levels may also improve mood, cognition and behavior in Parkinson’s disease patients [92]. Finally, vitamin D3 supplementation can reduce the risk and burden of COVID-19 consequences in PD patients [93].

## 5. Discussion

Neurodegenerative sequelae of infection—such as viral-associated Parkinsonism—have been previously addressed as “the third wave of the pandemic” [94]. This narrative review summarizes the current knowledge about the possible association between SARS-CoV-2 infection, frailty and Parkinson’s disease. The role of vitamin D and possible mechanisms of action that could link the three phenomena were considered. 

As PD patients age, their independence and quality of life decline, similar to those of frail elderly individuals; however, frailty and PD have different underlying pathologies [56,57]. Identifying long-COVID-associated frailty in patients with PD may have prognostic and therapeutic implications, since the diagnosis of frailty carries significance for quality of life, morbidity and life expectancy. 

It is reasonable to assume that an adequate vitamin D status reflects a balanced homeostasis and, therefore, promotes a more effective response to infection [95]. Conversely, homeostatic dysregulation by vitamin D is associated with frailty and neurodegeneration, whose progression or associated virus susceptibility was evident during the pandemic. Indeed, viruses have been implicated as causes or triggers of Parkinsonism for decades, but one of the most famous, albeit controversial, example is the 1918 von Economo encephalitis lethargica, a viral encephalopathy following the 1918 influenza pandemic [96]. In several sporadic cases (starting in subjects in their fourth to fifth decade), the Parkinsonian syndrome emerged within days or weeks following SARS-CoV-2 infection [97]. This observation was corroborated by abnormalities found on SPECT with DATSCAN or PET with fluorodopa. The evidence to date is insufficient to establish any causal link between SARS-CoV-2 infection and the subsequent development of Parkinsonism. On the contrary, the most credited hypothesis is that SARS-CoV-2 infection may “slatentize” the occurrence of Parkinsonism in the prodromal stage [97]. At the same time, low levels of vitamin D are associated with cognitive impairment and the development of Parkinson’s disease in its more severe form. Probably, an aging immune system and complex gene–environment interactions may contribute to the ‘perfect storm’ that enables PD to arise and progress [98]. 

**Figure 1:**
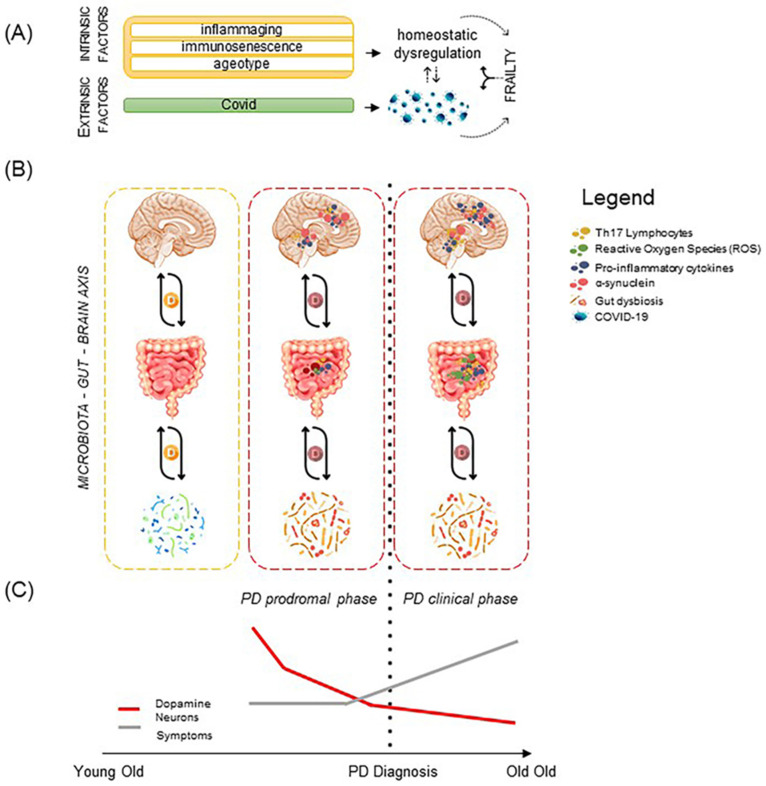
The complex interaction between inflammaging, immunosenescence and reduced microbiota diversity that accelerates the conversion from frailty to PD in the presence of SARS-CoV-2 infection is shown. The 3 panels should be read contextually. The pathological process is schematized in the continuum between the young–old (approximately 65–74 years of age) and the old–old (over age 85). Panel (**A**) Aging is influenced by multiple extrinsic and intrinsic factors leading to different phenotypes of the elderly. During the pandemic, multi-organ homeostatic dysregulation typical of old age was associated with the effects of viral infection, accelerating and exacerbating the processes of frailty. Frailty—in turn—contributed to aggravating both the consequences of COVID-19 infection and homeostatic dysregulation Panel (**B**) Overview of the microbiota-gut–brain axis related to the previous vicious circle that contribute to the PD occurrence. Panel (**C**) Schematic representation of the prodromal and clinical symptomatology of Parkinson’s disease over from adulthood to old age and the relative PD trajectories with respect to the relationship between dopaminergic cell depletion and manifest disease phenotype with reference to the microbiota-gut-brain axis alterations shown in panel (**B**). The current clinical diagnosis of PD is based on characteristic motor phenotypes that occur in advanced stages of the disease. These are preceded by prodromal symptomatology that manifests years to decades earlier, during middle age, but is less specific and varies greatly in timing and features among individuals.

**Figure 2:**
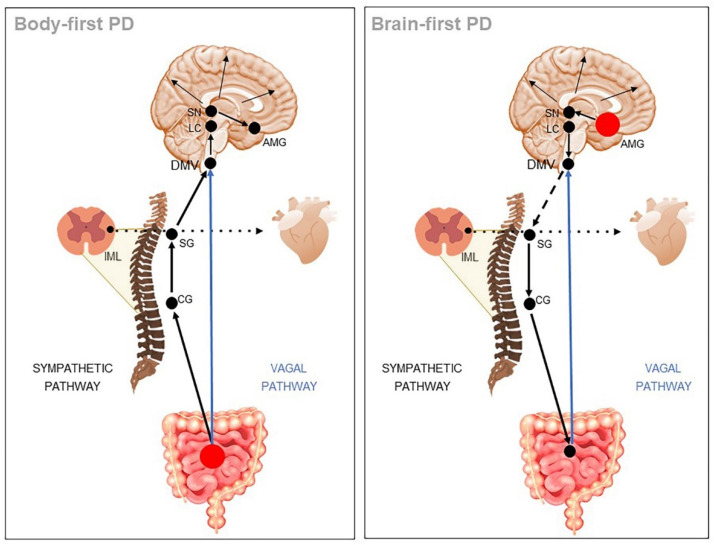
PD models based on *body-first* and *brain-first* spreading patterns are shown. According to the first pattern, α-synuclein may initially originate in the enteric nervous system before spreading to the brain. In the second case, it appears in the central nervous system and spreads to the brainstem and peripheral nervous system. In black “*sympathetic*”, in blue “*vagal*” pathways. Dotted vs. extended. DMV: dorsal motor nucleus of the vagus (brain stem); IML: intermediolateral nucleus (T1–L2, S2–S4); SG: stellate ganglion (C6–C7); CG: coeliac ganglion (T12–L1).

The whole process would seem to be associated with alterations in the microbiota–gut–brain axis (Figure 1), which fall squarely within the recent *α-Synuclein Origin site and Connectome (SOC) model*. The SOC model is a “vulnerability” model overlapping with frailty, which recognizes the significant role played by several factors, including inflammation, infection, microbiota, genetics, oxidative stress and calcium dysregulation [99]. Importantly, it identifies a *body-first PD*—which originates in the vitamin D-sensitive enteric nervous system—and a *brain-first PD*—closely connected with the COVID-19-sensitive olfactory bulb (Figure 2). The two subtypes differ on various clinical and imaging markers early in the disease course, whereas, as the disease progresses, the two phenotypes might converge [100]. This knowledge is important and challenges the understanding and treatment of prodromal and early stages of Parkinson’s disease that have been prevalent until now. 

The model also provides the impetus to investigate the mechanisms linking neuroinflammation and neurodegeneration, as well as the outcomes in terms of differential diagnosis. Neuroinflammation processes in PD involve multiple events, including activation of microglia and increased cytokine secretion [101]. The neutrophil-to-lymphocyte ratio (NLR) and the platelet-to-lymphocyte ratio (PLR) can be used as non-specific markers of inflammation in alpha-synucleinopathies in the clinical setting [101,102,103]. The NLR was derived from studies that showed that in chronic and acute diseases, the neutrophil rate tends to increase, while the lymphocyte rate tends to decline. An acute inflammatory or prothrombotic state can affect the PLR, which is a marker for changes in platelet and lymphocyte counts [102,103]. In PD patients, both biomarkers are significantly higher compared to healthy subjects. They may be useful in identifying PD Multiple-System Atrophy [103], as well as Progressive Supranuclear Palsy Syndrome and Corticobasal Syndrome [102].

In interpreting the results, several limitations of the studies that simultaneously addressed the relationship between COVID-19, frailty and PD should be considered. The data provided were aggregated to a high-level scale, so any reasoning about confounders was not possible. In addition, the observation period varied as regards waves and viral variants, with different COVID-19 incidence rates, as well as severity and type of symptoms and associated prognoses. Notably, information on COVID-19 patients with PD in the outpatient sectors or communities was not easy to find.

## 6. Conclusions

Currently, COVID-19 cannot be evaluated as a cause of an increased risk of Parkinsonism [6]. In addition to traditional pathological diagnoses, what additional information can virus-related neuroinflammation provide for Parkinsonisms? Hypovitaminosis D and SARS-CoV-2 infection’s high prevalence among European countries were associated based on speculative observations that do not verify or exclude the causal link. Yet, there is a strong relationship between SARS-CoV-2 infection, frailty, and Parkinson’s disease, as well as between vitamin D deficiency and all these conditions. 

PD patients are particularly recommended to use vaccination as a basic protection against SARS-CoV-2 infection [104], and the Movement Disorder Society has released a COVID-19 vaccine statement to address aspects of the COVID-19 vaccination among patients [105]. Approved mRNA-based vaccines and vector vaccines under development induce immunization through mechanisms that do not interact with the neurodegenerative process of PD. Regarding the inflammation associated with the disease pathogenesis, there is no evidence of any interaction with the immune response to these vaccines. Moreover, phase III data from clinical trials on the approved vaccines showed that the types or incidence of side effects in PD patients did not differ from those in the general population. Similar to reactions to other vaccinations, the COVID-19 vaccination does not interfere with the current PD therapies. Nevertheless, frail elderly have a pre-existing immunopathologic base that puts them at an increased risk of adverse outcomes and mortality due to COVID-19 and poor response to COVID-19 vaccination [2,42]. Moreover, their admission to Intensive Care Units (ICU) was based on their degree of frailty rather than their chronological age [42]. Consequently, it seems prudent to avoid administering the vaccine to elderly people with PD who are very frail and terminally ill [105]. This should especially be considered for cases of SARS-CoV-2 and influenza A co-infection. Indeed, most of those patients have comorbid conditions that reverberate with frailty [106,107]. Therefore, there is a need for a prompt identification of co-infection cases, especially during the flu season.

Misrecognizing the potential relationships between frailty and COVID-19 and between frailty and PD could result in uncertainty among ICU or vaccination workers as to whether to consider Parkinson’s disease as a “frail” condition, generating delays and poor patient care in some cases. To focus on the possible impact of physical and cognitive frailty could be fundamental in thinking about COVID-19 screening and management in PD. This can lead to personalized medicine protocols, balancing the long-term consequences of the pandemic.

## 7. Future Direction

To prevent, delay and more effectively treat PD, it is essential to harness the immunological knowledge emerging from PD patients to create better preclinical models. Studying how the immune system and frailty are linked to Parkinson’s disease may provide unprecedented opportunities to better understand the pathogenesis of the disease and identify clinically meaningful biomarkers and possibly even new therapeutic strategies. The use of anti-inflammatory agents might slow the progression of dopaminergic cell death in PD [101]. Importantly, Parkinsonism patients with anti-inflammatory treatment may also be less prone to dementia, a condition to which they are more susceptible [101]. 

In general, PD care can be improved by transforming the system of care toward an integrated network approach with increased interprofessional communication and the use of telemedicine while maintaining an attitude of preparedness in case of a pandemic. Because of the complex interplay between aging directions, disease, and environmental factors, the type of treatment will vary from patient to patient—and may need innovation in care, whether it be a new ICT-IoT or service, a drug cocktail, as well as possibly new neuromodulatory therapies [108,109].

## Data Availability

Not applicable.
